# Root architecture simulation improves the inference from seedling root phenotyping towards mature root systems

**DOI:** 10.1093/jxb/erw494

**Published:** 2017-02-07

**Authors:** Jiangsan Zhao, Gernot Bodner, Boris Rewald, Daniel Leitner, Kerstin A. Nagel, Alireza Nakhforoosh

**Affiliations:** 1 Department of Forest and Soil Sciences, University of Natural Resources and Life Sciences, Vienna (BOKU), Peter-Jordan-Straße 82, 1190 Vienna, Austria; 2 Division of Agronomy, Department of Crop Sciences, University of Natural Resources and Life Sciences, Vienna (BOKU), Konrad Lorenz-Straße 24, 3430 Tulln an der Donau, Austria; 3 Computational Science Center, University of Vienna, Oskar-Morgenstern-Platz 1, 1090 Vienna, Austria; 4 Institute of Biosciences and Geosciences, IBG-2: Plant Sciences, Forschungszentrum Jülich GmbH, D-52425 Jülich, Germany

**Keywords:** Lateral root branching, pea root system, phenotyping, root architecture model, root elongation rate, temporal scaling.

## Abstract

Root phenotyping provides trait information for plant breeding. A shortcoming of high-throughput root phenotyping is the limitation to seedling plants and failure to make inferences on mature root systems. We suggest root system architecture (RSA) models to predict mature root traits and overcome the inference problem. Sixteen pea genotypes were phenotyped in (i) seedling (Petri dishes) and (ii) mature (sand-filled columns) root phenotyping platforms. The RSA model RootBox was parameterized with seedling traits to simulate the fully developed root systems. Measured and modelled root length, first-order lateral number, and root distribution were compared to determine key traits for model-based prediction. No direct relationship in root traits (tap, lateral length, interbranch distance) was evident between phenotyping systems. RootBox significantly improved the inference over phenotyping platforms. Seedling plant tap and lateral root elongation rates and interbranch distance were sufficient model parameters to predict genotype ranking in total root length with an *R*_Spearman_ of 0.83. Parameterization including uneven lateral spacing via a scaling function substantially improved the prediction of architectures underlying the differently sized root systems. We conclude that RSA models can solve the inference problem of seedling root phenotyping. RSA models should be included in the phenotyping pipeline to provide reliable information on mature root systems to breeding research.

## Highlight

A root architecture model accurately predicts mature root systems of distinct pea genotypes from seedling stage phenotyping information of elongation rate and interbranch distance.

## Introduction

A decrease in the upward trend of yield since the 1990s is registered in major crops, including legumes. Among the suggested reasons are the increased frequency of environmental stresses ultimately limiting yield (Brisson *et al.*, 2010; Lobell *et al.*, 2011). Therefore, new approaches to improve crop performance are required, particularly for resource-limited environments (Rijsberman, 2006; MacDonald *et al.*, 2011). This is challenging considering the limited historic yield progress under stress conditions compared with improvements under high-yielding environments (Trethowan *et al.*, 2002). For three major legumes, Varshney *et al.* (2013) recently demonstrated that improvements for semi-arid environments have been very low over the last five decades.

Plant phenotyping is expected to facilitate crop improvement and stress resistance. Advances in imaging technology have led to high-throughput phenotyping platforms overcoming limitations in phenotypic data collection within conventional breeding (Passioura, 2012; Kuijken *et al.*, 2015). Current phenotyping platforms provide trait-based information on basic and secondary plant traits including root system architecture (Zhu *et al.*, 2011; Nagel *et al.*, 2012; Fiorani and Schurr, 2013; Granier and Vile, 2014; Fahlgren *et al.*, 2015).

In breeding, generally large numbers of candidate genotypes have to be screened to determine phenotypic variability in a given target trait. Therefore, breeders require phenotyping approaches with sufficient throughput to cope with large screening populations (Dhondt *et al.*, 2013). Sufficient throughput is particularly challenging when targeting the root system. Reviewing existing root phenotyping facilities, Zhu *et al.* (2011) conclude that ideally high throughput and resolution are combined. Most high-throughput root phenotyping is based on seedling plants grown on artificial media such as germination paper or agar (e.g. Bengough *et al.*, 2004; Nagel *et al.*, 2009; Christopher *et al.*, 2013; Le Marié *et al.*, 2014). Larger scale phenotyping systems for mature plants have been used to assess root traits related to superior drought resistance (e.g. Puangbut *et al.*, 2009; Gowda *et al.*, 2012). However, these set-ups are substantially less automatized and require a high amount of manpower for a comparatively restricted throughput.

A key question in phenotyping is whether observations are platform specific or can be transferred to other environments, particularly soil-grown plants (Wasson *et al.*, 2012). For example, Wojciechowski *et al.* (2009) investigated root traits of wheat genotypes in gel chambers, soil-filled columns, and field experiments, and demonstrated a significant influence of the experimental system on ranking among genotypes. In contrast, Watt *et al.* (2013) found significant correlations between root traits of wheat seedlings growing either on moist germination paper or *in situ*. Beyond the influence of the medium, extrapolation over time from seedlings towards mature root systems is questionable (Watt *et al.*, 2013). However overlapping quantitative trait loci (QTLs) of seedling root traits and crop yield (e.g. Tuberosa *et al.*, 2002) suggest that early-stage phenotyping should have some predictive value towards mature plants.

For monocot species, prediction of the mature root systems from seedling plants is difficult considering the dominance of a secondary shoot-borne root system that emerges at tillering (Hochholdinger, 2009; Zobel and Waisel, 2010). In contrast, dicots feature the main components of the root system (tap root and basal roots with their respective laterals) already at the seedling stage. Thus, the mature root system of dicots can be considered an extension of these structures with increasing axes length and branching order (e.g. Fitter and Stickland, 1992; Nielsen *et al.*, 1997).

According to Hodge *et al.* (2009), root systems can be described quantitatively by traits related to shape and structure. Several models on root system architecture (RSA) have been developed (Dunbabin *et al.*, 2013) to reproduce root system development from basic growth and branching rules and their modification by environmental stimuli (e.g. Pierret *et al.*, 2007; Leitner *et al.*, 2010). Until now, these models have been mainly used to assess root system functionality in terms of resource acquisition (e.g. water, Leitner *et al.*, 2014; phosphorus, Schnepf *et al.*, 2012) and underlying traits (e.g. distribution, Tron *et al.*, 2015; anatomy, Couvreur *et al.*, 2012, mycorrhiza: Schnepf *et al.*, 2008).

In this study, we suggest RSA models as a tool to overcome the inference problem of high-throughput phenotyping platforms. It is currently unknown to what extent root models can (i) reliably bridge between platforms differing in medium and accessed growing stage and (ii) improve predictions of breeding-relevant root traits of fully developed plants. We hypothesize that early-stage root traits from an agar-based phenotyping platform provide sufficient input parameters for model-based prediction of genotype ranking in mature root traits. Our study provides key root traits of pea (*Pisum sativum* L.) to be phenotyped for model-assisted extrapolation from seedlings towards mature RSA. The overall aim is to integrate RSA modelling into the phenotyping pipeline—allowing high-throughput phenotyping data to be translated into information relevant to breeding.

## Materials and methods

### Plant material

Sixteen cultivars of pea (*Pisum sativum* L.) were used for root phenotyping (Table 1). The cultivars originated from either Southern (Portugal and Spain) or Northern Europe (Estonia, Latvia, Norway, and Sweden) to represent the diversity of European pea cultivars; seeds were provided by partners within the EU FP7 Eurolegume project and by the Nordic gene bank.

**Table 1.** *Sixteen pea* (Pisum sativum *L*.) *cultivars used locally for food in different European countries and institutions donating the seeds for the experiment*

Abbreviation	Eurolegume number	Gene bank accession number	Local name	Country of origin	Donor institution ^*a*^
*Estonia1*	P58	EST2882	Eesti hall	Estonia	ECRI
*Estonia2*	P56	EST894	Eesti kollane söögihernes	Estonia	ECRI
*Estonia3*	P61	EST37	Jõgeva kirju	Estonia	ECRI
*Estonia4*	P65	EST41	Seko	Estonia	ECRI
*Latvia1*	P02		Alma	Latvia	SPPBI
*Latvia2*	P48		Bruno	Latvia	SPPBI
*Latvia3*	P12		k-4833 Stendes Hero	Latvia	SPPBI
*Latvia4*	P03		Retrija	Latvia	SPPBI
*Norway1*	P79	NGB10778	Aslaug	Norway	NordGen
*Norway2*	P82	NGB20045	Onkel Niels	Norway	NordGen
*Portugal1*	P53		Gp 3263	Portugal	INIAV
*Portugal2*	P51		Gp 3491	Portugal	INIAV
*Portugal3*	P52		Gp 3497	Portugal	INIAV
*Portugal4*	P54		Grisel	Portugal	INIAV
*Sweden1*	P90	NGB 102513	Svalöf Butter	Sweden	JTI
*Sweden2*	P88	NGB 13138	Odalett	Sweden	JTI

^*a*^ ECRI, Estonia Crop Research Institute; SPPBI, State Priekuli Plant Breeding Institute; NordGen, Nordic Genetic Resource Center; INIAV, Instituto Nacional de Investigação Agrária e Veterinária; JTI, Swedish Institute of Agricultural and Environmental Engineering.

### Root phenotyping experiments

Measurements of root traits were performed on two phenotyping platforms. One system represents a typical high-throughput phenotyping platform for seedling root screening using agar-filled plates. The other system focuses on mature root systems grown under more natural conditions (sand-filled columns) with less potential throughput.

#### Seedling root phenotyping

Root growth and architecture of seedling plants were monitored at the root phenotyping platform ‘GrowScreen-Agar’ at the Institute IBG-2: Plant Sciences, Forschungszentrum Jülich GmbH (Nagel *et al.*, 2009; Caliandro *et al.*, 2013). Pea seeds were surface sterilized with sodium hypochlorite and then sown on sterile agarose (1%, w/w) containing one-third modified Hoagland solution in Petri dishes (120 × 120 × 17 mm) as described previously (Nagel *et al.*, 2009). Seeds were pushed slightly into the sterile agar through a hole (diameter 5 mm, one seed per Petri dish) at one side of the otherwise sealed (Micropore, 3M Health Care, Neuss, Germany) Petri dishes. During germination, holes were covered with laboratory film (Parafilm, Pechiney Plastic Packaging) to keep the seeds moist. The Petri dishes were placed vertically in boxes to prevent light reaching the roots. In this way, the shoot could grow out through the hole, while roots grew inside the agar. Genotypes (16) were tested in 12 replicates, giving a total number of 192 Petri dishes which were transferred into a growth cabinet (Bioline VB 1100 Vario; Vötsch Industrietechnik, Germany) at 22/16 °C day/night temperature, 60% relative air humidity, a daylength of 16 h, and a light intensity of 140 µmol m^–2^ s^–1^ [phosynthetically active radiation (PAR)]. For imaging, Petri dishes were placed in the phenotyping platform ‘GrowScreen-Agar’ and images of each root system were taken automatically every second day via a high-resolution CCD camera (IPX-6 M3-TVM, Imperx Inc., Boca Raton, FL, USA). The experiment was stopped when the tap root reached the bottom of the Petri dishes between 7 d and 10 d after planting. Subsequently images were analysed using the software ‘GrowScreen-Root’ (Nagel *et al.*, 2009), providing information on root morphology (length of tap root and lateral roots) and RSA (number of lateral branches, branching angle representing the angle between the tap root and branched lateral roots).

#### Mature root phenotyping

Mature root phenotyping experiments were conducted in a plastic foil greenhouse located at the Institute of Agricultural Biotechnology of BOKU in Tulln, Austria (48.33°N, 16.05°E). Seeds of all cultivars were germinated in a growth chamber (Fitotron, Weiss-Gallenkamp, UK). Initial germination was conducted in darkness at 22/16 °C day/night temperature, 60% relative air humidity, and daylength of 16 h; after the first seed germinated, light was turned on with an intensity of 300 µmol m^–2^ s^–1^ (PAR). Seeds were coated with a rhizobium suspension (Steinberga *et al.*, 2008) before being planted in 0.5 litre plastic bags (10 cm high) filled with washed quartz sand (0.7–1.2 mm in siz) amended with 1 g of slow-release fertilizer (Osmocote Pro 3-4M, Everris Int., The Netherlands). Seven to eight days after germination (DAG), eight similar-sized seedlings per cultivar were selected for transplanting. In the greenhouse, eight blocks of 16 plastic tubes (genotypes) each were established in a complete randomized block design. The plastic tubes used as pots/growing cylinders were 108 cm long and 20 cm in diameter (~32 litres); the bottom was sealed with a cap; holes covered with a glass fibre mat allowed for free drainage. Before tubes were filled with washed, 0.7–1.2 mm sized quartz sand, a plastic liner was installed in each tube allowing for undisturbed removal of the whole substrate during harvest; the liner was perforated at the bottom 10 cm. Then 8.3 g of an AMF inoculum (*Glomus mosseae* BEG95, *G. intraradices*, and *G. geosporum* BEG199; supplied by Dr Aleš Látr, Symbiom, Czech Republic) were added to each plant individual around the root systems at depths of 0–10 cm before the tube was filled to the brim with additional sand. The symbiont inoculation (rhizobia+AMF) approximates microbial-mediated nutrient acquisition in field environments which can influence the shape of root systems (Li *et al.*, 2016). An automated, pressure-compensated drip-irrigation system was used to supply all plants with ample amounts of water and a modified Long Ashton nutrient solution (Jia *et al.*, 2004); amounts were adjusted to increasing plant size and weather conditions. Numbers of flowers per plant were counted every other day after observing the first flower. Plants were harvested in blocks at 82 ± 2 d after transplanting at the end of flowering (BBCH 69–71) when the root system is considered to be fully developed (Thorup-Kristensen, 1998). After harvesting the shoots (data not shown), the plastic tubes were placed horizontally and the plastic liner was pulled out on a 1.5 mm mesh table. After cutting the plastic liner open, roots were manually excavated as described by Kashiwagi *et al.* (2005) and others. No roots reached the bottom of the tube and few roots were discovered at the sides, indicating a rather unrestricting pot size. The uncovered root system was washed and rinsed in a bucket filled with clean tap water, photographed, and stored in a water-filled plastic bag at 4 °C until further analysis (1–3 weeks) took place (Hu *et al.*, 2013). For in-depth architectural and morphological analysis, the root systems of 5–7 plant individuals per cultivar were manually dissected into tap root and laterals. Laterals along the tap root and the tap root were separated into the six depth classes 0–2.5, 2.5–5, 5–10, 10–20, 20–40, and 40–100 cm. The number of lateral roots from each depth was counted. Subsamples of lateral roots were scanned in water-filled trays (Epson Expression 10000XL; Epson, Japan) at 400 dpi, greyscale. Pictures were analysed for diameter, surface area, length, and volume with the PC program WinRhizo 2012b Pro (Régent Inst., Quebec, Canada).

### Root simulation

#### Model description

**Fig. 1.**
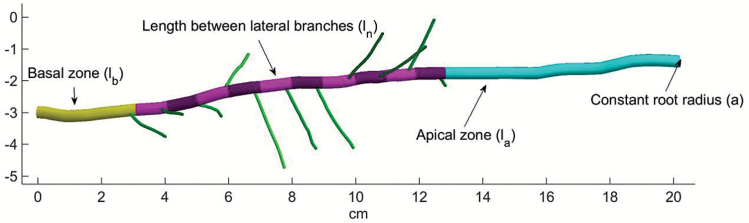
Basic structure of simulated root axes. Basal zone, branching zone, and apical zone of a simulated root. First-order laterals are also depicted. (This figure is available in colour at *JXB* online.)

The root architecture model RootBox (Leitner *et al.*, 2010) describes the growth of individual root axes and their laterals. Each root consists of a basal, a branching, and an apical zone (Fig. 1).

The root elongates according to negative exponential growth; that is, the length *l* of the root at a certain time *t* is given by

$$l\left(t\right)=k(1-e^{-\frac{r}{k}t})$$ (1)

where *k* is the maximal length the root will reach, and *r* is the initial growth rate. After the basal zone and the apical zone have developed, lateral roots start to emerge at a fixed branching angle θ within the developing branching zone. The maximal length *k* of a root is given by

$$k=l_{\text{a}}+l_{\text{b}}+\left(nob–\text{1}\right)l_{\text{n}}$$ (2)

where *l*_a_ is the length of the apical zone, *l*_b_ is the length of the basal zone, *l*_n_ is the interbranching distance, and *nob* is the maximal number of lateral branches the root will develop. All parameters are given by mean and SD.

The direction of root growth is determined according to a random optimization process: from *N* small changes in root tip direction the one is chosen that best suits an objective function. This function describes the type of tropism used (e.g. an objective function describing gravitropism picks directional changes downwards and another one describing hydrotropism favours changes towards higher water content). Tropism is described by three parameters: type (defines the objective function), *N* (the number of trials), and σ (strength of changes in root direction).

#### Model parameterization

Simulation of a plant root system requires setting the parameters for each root order. Table 2 gives a list of parameters and the respective values used for simulation of the pea Supplementary Protocol S1 at *JXB* online.

**Table 2.** Model parameters for simulation of pea root systems (*m* measured values)

Root trait class	Parameter Description	Parameter abbreviation	Unit	Tap root	First- order lateral	Second-order lateral
Growth	Initial elongation rate	*r*	cm d^–1^	*m* (see Table 5)	*m* (seeTable 5)	Equal first-order *r*
	Growth function	*gf*	{1,2}^*a*^	See scenarios	See scenarios	See scenarios
	Phenological constraint	*sef*	–	See scenarios	See scenarios	See scenarios
Morphology	Length of basal zone	*l*_a_	cm	0.5	0.5	0.5
	Length of apical zone	*l*_b_	cm	2.5	2.5	–
	Length between lateral branches	*l*_n_	cm	*m* (see Table 4)	Equal tap root *l*_n_	–
	Maximal number of branches	*nob*	Number	See scenarios	See scenarios	See scenarios
Spatial arrangement	Distribution of interbranch distance	*sbf*	–	See scenarios	1	1
	Branching angle	θ	rad	–	1.05 (60°)^*c*^	1.57 (90°)^*c*^
	Tropism type	*type*	{0,1,2,3}^*b*^	1	1	1
	Tropism strength	*N*	1	1.5	1	1
	Root flexibility	σ	cm^–1^	0.3^*d*^	0.3^*d*^	0.3^*d*^
Biomass	Root radius	*a*	cm	0.07	0.04	0.02
	Root life span	*rlt*	days	inf.	inf.	inf.

^*a*^ 1 is exponential rise to maximum, 2 is linear growth.

^*b*^ 0 is no tropism, 1 is gravitropism, 2 is hydrotropism, and 3 is chemotropism.

^*c*^ Mean of measured root angles in the seedling root phenotyping platform.

^*d*^ Default values used according to Leitner *et al.* (2010).

The growth function (*gf*) determines the type (linear, exponential) of elongation with the parameter *r* driving the elongation rate (see Equation 1 for the exponential case) and *sef* (scale elongation function) allowing for a growth reduction scaling function. Each root axis is composed of an unbranched basal (*l*_b_) and apical (*l*_a_) zone and the branching zone with laterals emerging at the set distance (*l*_n_). The maximum number of branches (*nob*) is generally set to a high number; thereby the actual number of laterals is the result of the final length of a root axes obtained from the elongation function and the interbranch distance (*l*_n_). The spatial arrangement of root axes in a given volume is determined by the insertion angle of branches on their mother axes (θ) and different types of tropism (Leitner *et al.*, 2010). Root flexibility is an empirical parameter incorporating soil mechanical and plant physiological causes of tip deflection upon root penetration through the soil. Root biomass is not explicitly accounted for by the model. Indirectly it is related to the model parameters for axis thickness (root radius *a*) and root life span (*rlt*).

**Fig. 2.**
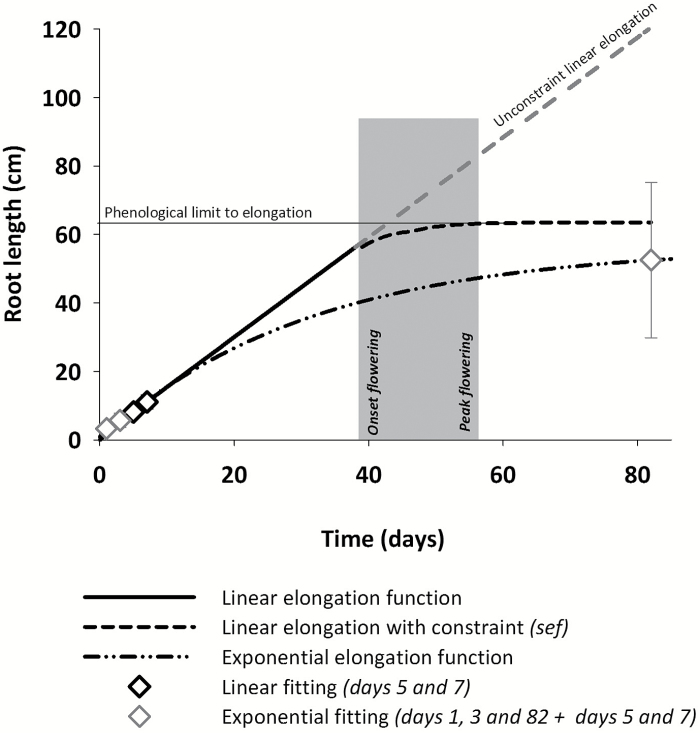
Example of curve fitting to obtain the root elongation rate parameter (*r*) for the linear and exponential growth models. The linear growth is constraint to an upper limit by the time of flowering when further elongation is stopped. The grey-shaded area shows the time span between onset and peak flowering used in the elongation scaling function (*sef*) for a smooth decrease of root elongation to zero.

Our study uses the model to predict the ranking among genotypes in terms of root length, number of lateral branches, and root distribution in mature root systems. The key parameters driving root length as a global morphological descriptor are growth (parameters *r* and *gf*) and branching frequency (parameter *l*_n_). The lengths of the non-branched basal and apical zones were set constant for all genotypes due to the lack of data and their minor influence on the final result. Two types of growth functions *gf* were tested: (i) linear growth (Scenarios 1 and 3) and (ii) exponential rise to maximum (Scenario 2). For the linear growth, we used the slope of a linear regression fit through the two last measured time points (DAG 5 and 7) of the seedling root data before the tap root reached the bottom of Petri dishes (Fig. 2).

The tap root in particular showed a decreasing elongation rate within 1 week of measurement in the seedling phenotyping system: the growth rate between days 5 and 7 was 11.6% (6.4%) lower compared with the rate between days 3 and 5 (1 and 3). Thus selecting the final two data points of the seedling experiment is considered as reducing the risk of overestimating root elongation at initial stages. For the laterals, the length of an average single lateral root was calculated by dividing the total length of laterals by their number. A linear elongation function was fitted in Scenarios 1 and 3 to this lateral root of average length using the final two data points of the Petri dish experiment to reduce a possible bias from quick initial elongation rates.

**Fig. 3.**
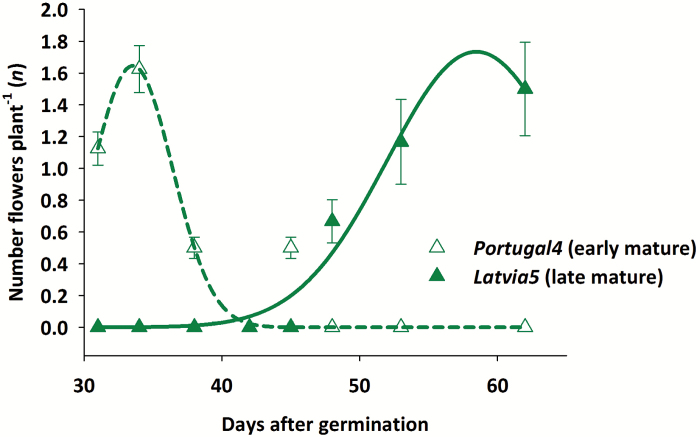
Example for determination of peak time of flowering for an early and a late mature genotype with a three-parameter Gaussian curve (measurement points show the mean ±SD). (This figure is available in colour at *JXB* online.)

If linear growth was assumed (Scenarios 1 and 3), a new growth reduction function (*sef*), restricting elongation from a given time point onwards, was defined. The function assumes a linear decrease in growth rate over a certain time span until growth stops (Fig. 2). We used the appearance of first flowers as the starting time point and peak flowering as the time where root elongation stops (see Fig. 3). The maximum number of laterals along the branching zone (*nob*) was set to 1000 in Scenarios 1 and 3; this allows for a theoretically unconstrained root elongation—limited only by the growth reduction function.

In the case of an exponential elongation rising to a maximum in Scenario 2, the function was parameterized including the initial growth and the final data point as maximum length (Fig. 2). In this case, *nob* had to be set to a value that restricts maximum root length to the measured target values of the mature root systems. Using the exponential function assumes that including the final root length results in an optimum fit of the growth pattern. Thereby we could compare to what extent the goodness of fit between measured and simulated root length changed compared with a simulation based on a linear growth rate from seedling root observations combined with phenological constraints only. This comparison should indicate whether initial growth information obtained by seedling phenotyping platforms is sufficient for an accurate prediction or if additional data points at later ontogenetic stages (with the final length as the hypothetical optimum information) are required for an appropriate parameterization of the growth function.

Beyond assessing prediction of the overall root system size (root length), we also compared two architectural descriptors, namely (i) number of first order laterals and (ii) distribution of root length along the tap root. A high root length can be achieved either by high elongation rates or by high branching frequency. In the simulation scenarios, the number of first-order lateral roots along the tap root is the product of parameter values for interbranch distance (*l*_n_) and tap length resulting from the applied elongation functions.

Root length distribution over depth is essential to capture the capacity of cultivars with distinct root architectures to explore soil resources. It is a result of lateral branching frequency at different segments of the tap root, the angle of emergence between tap and lateral roots, and tropism influencing growth direction. For Scenarios 1 and 2, a constant branching frequency (*l*_n_) from seedling root phenotyping was used, while for Scenario 3 a new scaling function for branching frequency (*sbf*) was implemented. This function modifies *l*_n_ in a given tap root segment according to a set probability; for example, an *sbf* of 0.5 means that for *l*_n_ equal to 0.1 (i.e. 10 laterals cm^–1^ tap root) the probability of emergence is only half (i.e. 5 laterals cm^–1^). Parameterization of *sbf* was based on mature root phenotyping data of lateral numbers in different tap root segments (base to 2.5 cm, 2.5–5 cm, 5–10 cm, 10–20 cm, 20–40 cm, and 40 cm to apex).

Comparison of root angles and growth direction was not feasible due to the *ex situ* measurements in the mature root phenotyping system that essentially change these two parameters of spatial root arrangement. Therefore, constant values were used for branching angle (average of measured angles in Petri dishes), tropism strength, and root tip flexibility (default values from Leitner *et al.*, 2010). Also for parameters related to root biomass and decay (radius, life span), fixed values were used or they were considered as not restricted.

#### Simulation scenarios

Three simulation scenarios were used to determine critical parameters for achieving a reliable model-based up-scaling from seedling to mature pea root systems. Table 3 provides an overview on the tested scenarios and the respective measurements included in the parameterization.

**Table 3.** Simulation scenarios and measurement information required for parameterization

Scenario	Relevance	Measurement information ^*a*^	
		Seedling root system	Mature root system
1	Prediction based on (i) seedling root data+phenological constraint for elongation by flowering information and (ii) seedling root data for branching.	*r*_tap_ *r*_1st order_ *l*_n,tap_	Flowering
2	Prediction based on (i) seedling+final root data for elongation with exponential rise to maximum without phenological constraint by flowering information and (ii) seedling root data for branching.	*r*_tap_ *r*_1st order_ *l*_n,tap_	*r*_tap_ *r*_1st order_
3	Prediction based on (i) seedling root data+phenological constraint for elongation by flowering information and (ii) unevenly spaced final root data for branching.	*r*_tap_ *r*_1st order_	*l*_n,tap_+*sbf* Flowering

^*a*^ *r*_tap_ is the elongation rate of the tap root; *r*_1st order_ is the elongation rate of the first-order laterals branched from a tap root; *l*_n,tap_ is the length between lateral branches along the tap root, and *sbf* is the scaling function for uneven spacing (cf. Table 5 for average and first segment *l*_n_); flowering refers to measured times of onset and peak flowering (cf. Table 4).

The first scenario relies on seedling root data only. Root elongation is constrained by phenological information related to the onset and duration of flowering (i.e. a quantification of earliness of the respective genotypes).

The second scenario uses final root lengths—assuming that exponential elongation towards measured, final values (as constraints for maximum root length) represents the theoretical optimum for reproducing the observed root growth. This scenario thereby determines the relevance of improved information beyond seedling root data (i.e. longer phenotyping duration) to approximate root growth realistically.

Similar to Scenario 1, the third scenario simulates root elongation based on seedling data and phenological constraints. However, here the relevance of data on root branching acquired from more advanced stages (mature plants) is tested—using the final branching frequency data as the theoretically optimum information to parameterize a branch scaling function (*sbf*).

### Statistical evaluation

Empirical root data are evaluated by ANOVA using a generalized linear model (SAS Version 9.4, SAS Institute, Inc., Cary, NC, USA; procedure PROC GLM). Parameterization of the growth functions was conducted using non-linear fitting (SAS procedure PROC NLIN) with a Marquardt optimization algorithm. Plant breeders’ decision-making on genotypes to be selected for further breeding due to their superiority in a given target trait is generally based on ranking within the test population. Therefore, the Spearman rank correlation coefficient is used to evaluate the accuracy of simulated root traits to predict the observed ranking among genotypes. Beyond the rank order, simulation results are also evaluated for accurate prediction of absolute values of observations. For this, Bellocchi *et al.* (2010) recommended a combination of different evaluation statistics to ensure an unbiased judgement of the simulation quality. Difference-based statistical goodness of fit indicators [root mean square error (RSME), percentage mean error, and index of agreement] were calculated by the software IRENE v.1.0 (Fila *et al.*, 2003). Regression (SAS procedure PROC REG) is used to evaluate the departure of predictions from a hypothetical optimum agreement with zero intercept and the slope following the 1:1 line (for slope comparison, see Sawand, 2012).

## Results

### Flowering phenology

The pea accessions investigated are from different origins with diverse whole-plant characteristics. Here we only report the different phenology, evidenced by the times until onset of flowering and peak flowering. Flowering pattern and related changes in root–shoot assimilate sink provide the quantitative descriptor of earliness used for a root elongation reduction function. The data were obtained by fitting a Gaussian peak function to the number of flowers counted on each plant in the greenhouse; Fig. 3 exemplifies the procedure for an early- and late-flowering genotype, respectively.

The day of the first flower, peak flowering, as well as the quality of fitting are reported in Table 4. The early-flowering genotypes (*Estonia4*, *Norway2*, *Portugal1*, *Portugal4*, and *Sweden2*) started flowering at DAG 31. The latest genotype to flower was *Latvia4* with the onset of flowers at DAG 48. Florescence was longest for genotypes *Estonia2* and *Sweden1*, while the shortest florescence was noted for *Portugal3* and *Portugal4*.

**Table 4.** Start and peak time of flowering of pea genotypes (peak flowering based on fitted three-parameter Gaussian curve, cf. Fig. 3) at days after germination (DAG) as observed in the mature plant phenotyping platform

Genotype	Start flowering	Peak flowering	Fitting parameters	
	DAG	DAG	*R* ^2^	*P*-value
*Estonia1*	34	41.3	0.97	0.0009
*Estonia2*	34	49.1	0.70	0.0488
*Estonia3*	38	44.3	0.93	0.0045
*Estonia4*	31	36.7	0.82	0.0143
*Latvia1*	48	58.5	0.97	0.0002
*Latvia2*	42	50.8	0.76	0.0547
*Latvia3*	42	55.8	0.93	0.0014
*Latvia4*	38	50.6	0.76	0.0288
*Norway1*	42	50.3	0.73	0.0370
*Norway2*	31	41.4	0.98	<0.0001
*Portugal1*	31	37.8	0.70	0.0276
*Portugal2*	34	40.8	0.71	0.0459
*Portugal3*	38	41.6	0.97	0.0002
*Portugal4*	31	33.5	0.91	0.0027
*Sweden1*	34	53.1	0.70	0.0502
*Sweden2*	31	38.0	0.72	0.0419

### Root phenotyping traits

Root traits had a rather high variability, with coefficient of variation (CV) ranging from 77.7% (lateral root length; mature root systems) to 26.4% (tap root length; seedling root systems). Generally the CV was higher in the mature root phenotyping platform compared with the seedling root platform. Lateral root length was most variable, while tap root length had the lowest CV in both platforms.

The statistical evaluation of measured root traits characterizing length and branching demonstrates significant interaction between phenotyping platforms and genotype (Table 5). This points to changing ranks of genotypes depending on phenotyping conditions and indicates that prediction of larger mature root systems from short-term seedling root observation is problematic. For example, in the seedling phenotyping platform, genotype *Sweden1* had the longest tap and lateral roots, while in the mature phenotyping platform genotype *Latvia3* featured the longest tap root and genotype *Latvia4* had the longest total length of lateral roots. Similarly, *Estonia1* seedlings possessed the densest lateral branching (i.e. lowest interbranch distance along the tap root), while at maturity genotype *Latvia4* showed the lowest lateral root branching distance. Interbranch distance decreases acropedally with a lateral branch distance in the first tap root segment (base to 2.5 cm) of only 32.3% (seedling) and 23.4% (mature) compared with the average. Detailed interbranch distances in six intervals along the taproot are provided in Supplementary Table S1.

**Table 5.** Tap/lateral root length and distance between laterals along the tap root (average and first segment 0–2.5 cm) of seedling and mature pea root systems

Genotype	Tap root length (cm)		Lateral root length (cm)		Interbranch distance ^*a*^ *(cm* )			
	*Seedling*	*Mature*	*Seedling*	*Mature*	*Seedling*		*Mature*	
*Estonia1*	9.1	44.7	0.1	782.9	0.34	(0.14)	0.80	(0.19)
*Estonia2*	12.8	70.4	97.1	3114.2	0.54	(0.17)	0.70	(0.14)
*Estonia3*	7.7	74.7	51.2	1326.4	0.71	(0.30)	1.07	(0.13)
*Estonia4*	11.6	47.0	57.6	623.2	0.70	(0.18)	0.94	(0.26)
*Latvia1*	11.5	63.3	73.9	4072.7	0.58	(0.19)	0.80	(0.22)
*Latvia2*	10.9	36.4	69.5	3069.7	0.72	(0.18)	0.60	(0.13)
*Latvia3*	8.8	79.9	44.4	2872.4	0.59	(0.19)	0.71	(0.23)
*Latvia4*	11.1	52.5	96.6	5080.4	0.41	(0.11)	0.37	(0.11)
*Norway1*	8.5	49.8	46.4	2354.1	0.37	(0.12)	0.44	(0.12)
*Norway2*	6.6	58.0	34.4	2462.0	0.38	(0.17)	0.78	(0.12)
*Portugal1*	8.9	49.0	69.4	1685.2	0.35	(0.11)	0.73	(0.13)
*Portugal2*	8.5	46.8	51.8	1817.6	0.52	(0.16)	0.70	(0.17)
*Portugal3*	9.5	40.4	42.7	2260.7	0.69	(0.24)	0.51	(0.11)
*Portugal4*	10.8	77.4	65.0	764.8	0.51	(0.14)	0.96	(0.15)
*Sweden1*	13.5	49.8	120.2	4699.0	0.47	(0.12)	0.72	(0.21)
*Sweden2*	9.5	49.7	48.3	277.9	0.67	(0.24)	1.80	(0.53)
SED	0.3	3.3	2.9	179.5	0.02	0.003	0.05	0.05
CV (%)	26.4	49.2	36.6	77.7	31.2	42.1	50.5	41.8
G	0.013		<0.001		<0.001 (<0.001)			
PLATFORM <0.001			<0.001		<0.001 (<0.001)			
G×PLATFORM 0.009			<0.001		0.018 (<0.001)			

Means are reported; SED is standard error of differences, CV is coefficient of variation. Significance level of main effects (G, genotype; PLATFORM, phenotyping platform) and interactions is reported by the *P*-value.

^*a*^ Average (total number of first-order laterals divided by tap root length) and smallest (in parenthes; base to 2.5 cm to tap root) interbranch distances. More detailed data in six intervals along the tap root for parameterization of the branch scaling function *sbf* are given in Supplementary Table S1.

**Fig. 4.**
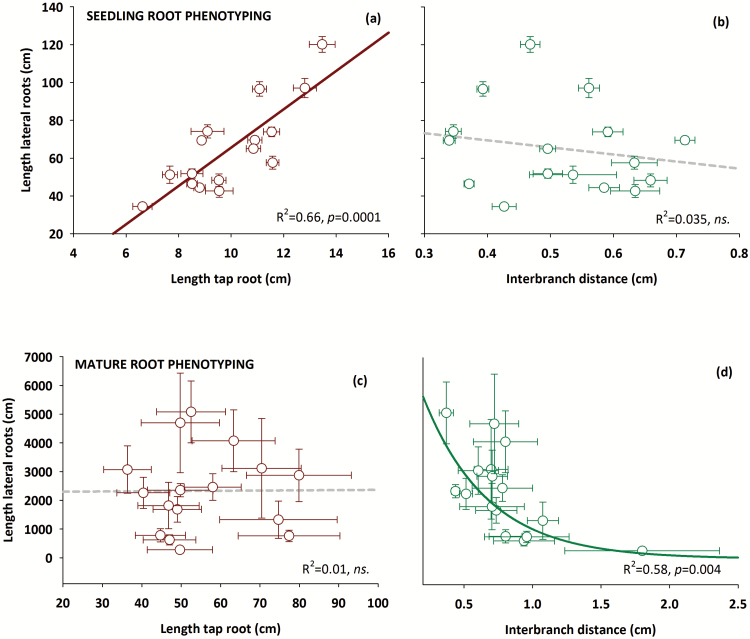
Changing drivers of total lateral root length at different stages of root system development (a, b seedling root phenotyping; c, d mature root phenotyping). (a and c) The influence of tap root length. (b and d) The influence of interbranch distance along the tap root (means ±SD). (This figure is available in colour at *JXB* online.)

Interestingly there is a change in the role of tap root length versus interbranch distance in determining total lateral length (Fig. 4). At early-stage phenotyping, distinct elongation rates of the tap root obviously have constrained the length of laterals among genotypes, while there is still no significant influence of interbranch distance. In contrast, our results suggest that for the mature root system the main factor for differences in total lateral root length among genotypes is the interbranch distance—the frequency of branching points along the tap root where laterals emerge—while the total tap root length has no effect.

### Relationships between phenotyping platforms

**Fig. 5.**
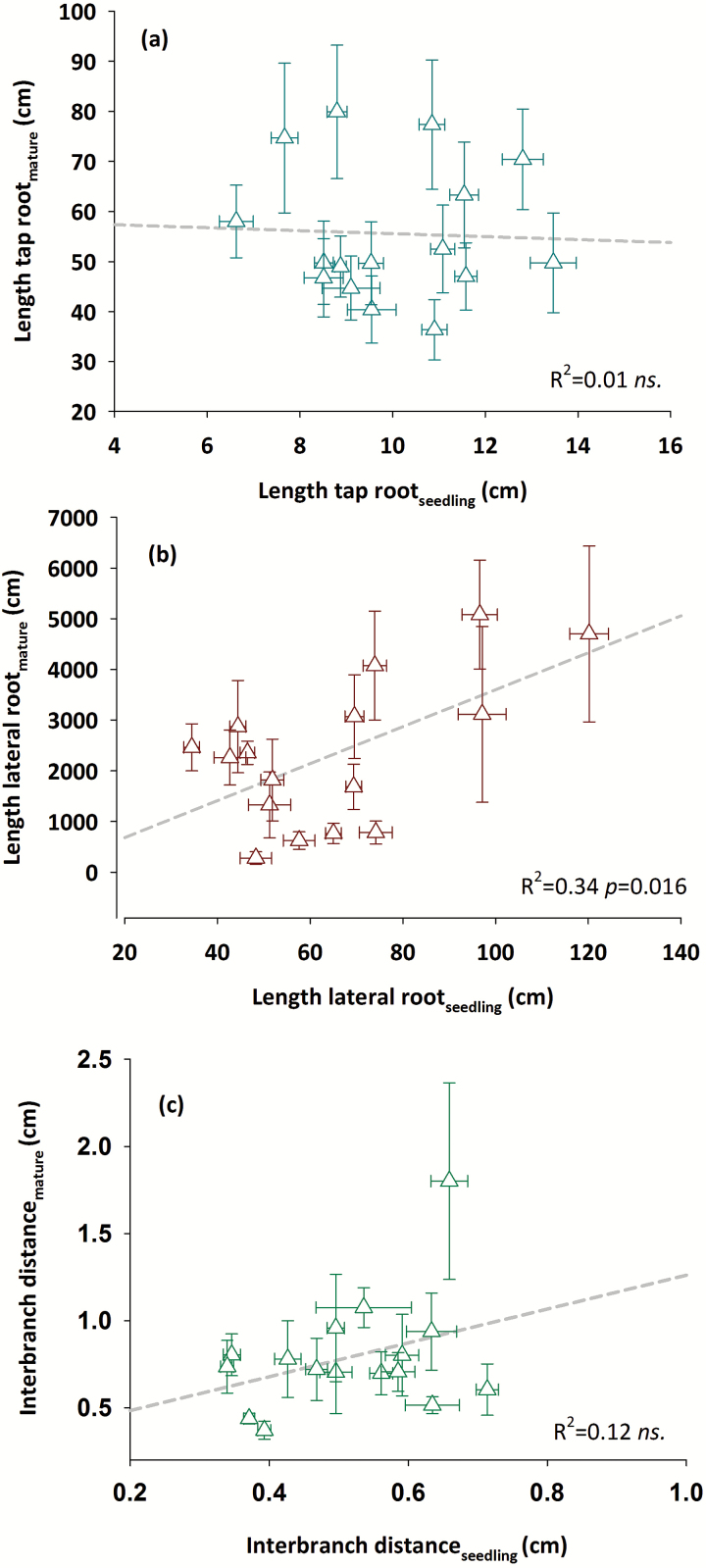
Relationship between root morphological (a, length of tap root; b, length of lateral root) and branching traits (c, interbranch distance) measured at seedling (agar-based phenotyping root platform; 7 d after germination) and post-flowering stage (large sand-filled column phenotyping system; 82 ± 2 d after germination). (This figure is available in colour at *JXB* online.)

The essential question arising is whether parameters obtained in early-stage, Petri dish-based phenotyping platforms can be related directly to traits of mature root systems by any empirical function. The significant G×PLATFORM interaction in Table 5 points to platform-specific expression of traits. Figure 5 depicts the linear regression between common parameters measured at both phenotyping platforms. It is evident that there is only a very weak relationship among root systems characterized at the seedling and mature stage (highest *R*^2^ = 0.34 for lateral root length), suggesting that direct inference on larger versus small mature root systems from an early-stage phenotyping platform is not reliable. The data also do not show evidence of any other non-linear function to relate the two phenotyping situations.

### Model application

#### Measurement-derived simulation parameters

The growth parameters for the three simulation scenarios calculated from measured root lengths of tap root and laterals (see Fig. 2) are given in Table 6. Interbranch distance *l*_n_ is directly taken from measured values (Table 5), while the phenological constraints for the root elongation reduction function (*sef*) is derived from the observed initiation of flowering and peak flowering dates (Table 4).

**Table 6.** Parameters driving root elongation in the linear and exponential simulation scenarios calculated from phenotyping observations The parameter *r* gives growth rates in cm d^–1^ for the tap (*r*_tap_) and the lateral roots (*r*_lateral_). *nob* is the maximum number of laterals emerging along the branching zone of the tap (*nob*_tap_) and lateral roots (*nob*_lateral_).

Genotype	Linear (Scenario 1, 3)^*a*^		Exponential (Scenario 2)			
	*r*_tap_	*r*_lateral_	*r*_tap_	*r*_lateral_	*nob*_tap_	*nob*_lateral_
*Estonia1*	0.59	0.64	1.65	0.36	131.5	13.0
*Estonia2*	1.30	0.89	2.25	0.48	130.4	19.5
*Estonia3*	1.25	0.62	1.10	0.35	105.2	18.7
*Estonia4*	0.97	0.70	2.03	0.38	67.1	10.6
*Latvia1*	1.08	0.86	1.95	0.41	109.1	30.0
*Latvia2*	1.14	1.05	1.92	0.54	50.6	41.3
*Latvia3*	1.07	0.64	1.34	0.35	135.4	24.2
*Latvia4*	1.46	0.81	1.82	0.36	128.0	35.0
*Norway1*	1.28	0.42	1.31	0.26	134.6	20.8
*Norway2*	0.85	0.43	1.03	0.38	152.6	31.3
*Portugal1*	0.51	0.59	1.65	0.35	140.0	22.7
*Portugal2*	0.95	0.69	1.39	0.38	90.0	23.2
*Portugal3*	1.18	0.66	1.62	0.27	58.6	26.8
*Portugal4*	0.77	0.69	1.94	0.39	151.8	8.4
*Sweden1*	1.20	0.92	2.50	0.52	106.0	38.7
*Sweden2*	0.97	0.77	1.57	0.43	74.2	7.0

^*a*^ For Scenarios 1 and 3 the maximum number of branches (*nob*) is set to 1000, i.e. no fixed constraint on branching number is used.

For the linear elongation function *gf* (Scenarios 1 and 3), a large variation in both tap and lateral root elongation rates occurred, ranging from 0.51 cm d^–1^ (*Portugal1*) to 1.30 cm d^–1^ (*Estonia2*) for the tap root, and from 0.42 cm d^–1^ (*Norway1*) to 1.05 cm d^–1^ (*Latvia2*) for lateral roots.

In the exponential root elongation scenario (Scenario 2), root growth continued until the measured root length of mature plants was reached. Thus, the model requires setting *nob* to a given value calculated by dividing the measured length by the interbranch distance (see Table 5). In this scenario, genotype *Sweden1* has the highest tap root growth rate while *Norway2* has the slowest increase towards the pre-set maximum length. Exponential growth rates of laterals are greatest/smallest for genotypes *Latvia2* and *Norway1*, respectively. For lateral roots, both growth models result in similar ranking among genotypes (*R*^2^=0.66, *P*<0.001); this, however, is not the case between tap root growth models (*R*^2^=0.02, *P*=0.579)

**Fig. 6.**
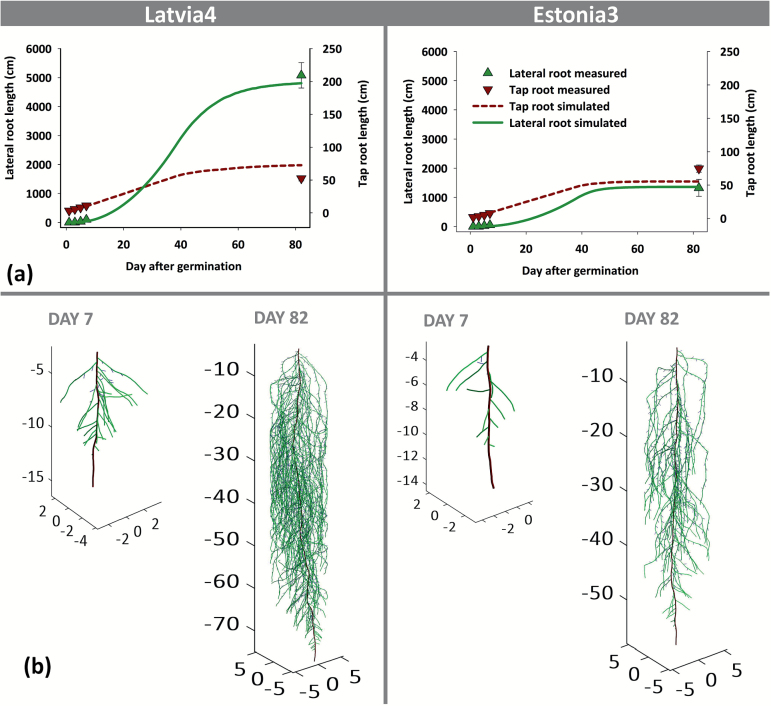
Measured and simulated root growth for two pea cultivars differing in total root length (left, Latvia4 representing a dense rooting genotype; right, Estonia3 representing a sparsely rooted genotype). (a) Measured (DAG 1–7 in agar-based platform; DAG 82 in sand-column platform) and simulated tap and lateral roots; (b) Simulated root system architectures of the genotypes at early stage (DAY 7) and full root development (DAY 82), simulated with Scenario 1 (cf. Table 3). We notice that for lateral root elongation parameters, the average length of single laterals (cf. the Materials and Methods) was used, while the figure shows the total lateral length. (This figure is available in colour at *JXB* online.)

Figure 6 provides a graphical example for two contrasting pea genotypes (large versus small root system). The figure illustrates the measured data points used for deriving the respective root elongation parameters, modelled growth curves for both tap and lateral root length, as well as the resulting simulated seedling (DAG 7) and mature (DAG 82) root system architectures.

#### Simulation-based prediction of mature root length

**Fig. 7.**
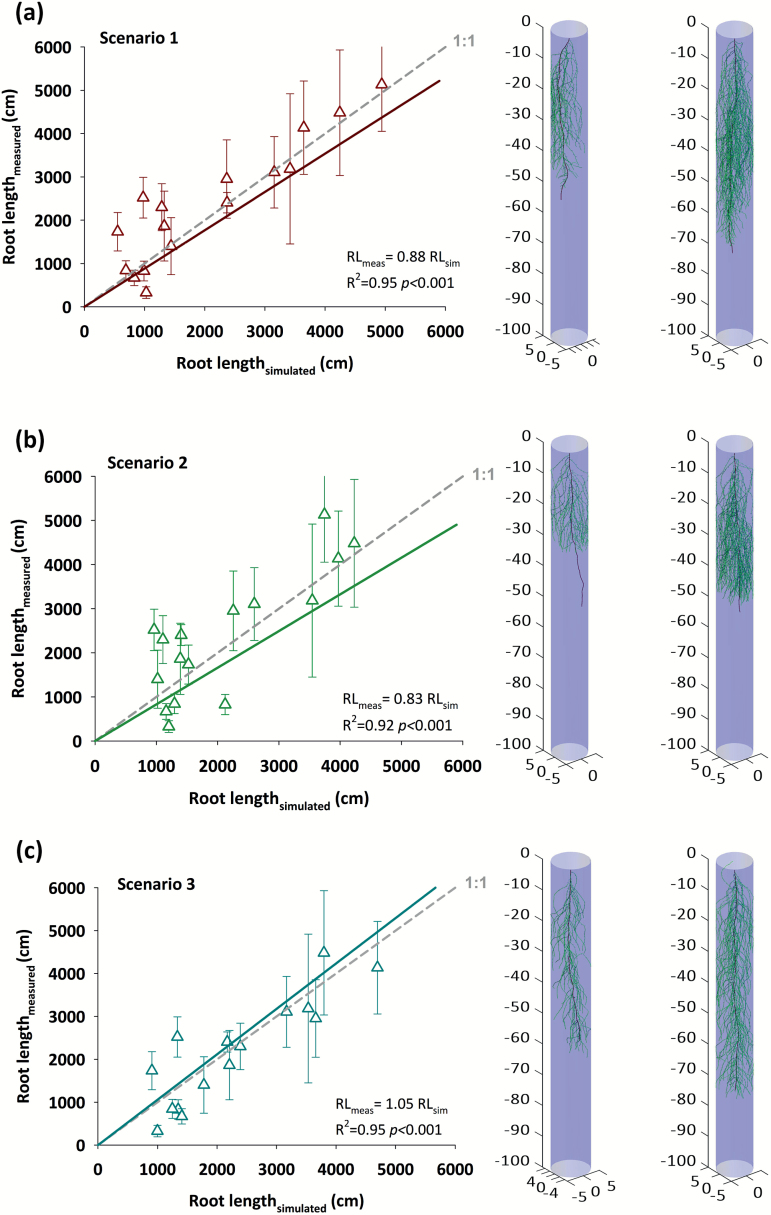
Prediction of measured total root length using the RSA model RootBox calibrated with (a) early-stage phenotyping data only and phenology as root elongation constraint, (b) including final tap and lateral root length for root elongation parameterization and branching frequency of laterals along the tap root at seedling stage, and (c) early-stage phenotyping data and phenology constraint for root elongation and uneven final branching frequency of laterals along the tap root. The left side shows the linear relationship (regression with zero intercept) between simulated and measured (mean ±SD) root length *R*^2^, slope parameter and significance (*P*-value). At the right, example images of simulated root systems with short (*Estonia3*) vs. long (*Latvia4*) total root length are provided. (This figure is available in colour at *JXB* online.)

Figure 7 shows the prediction of measured root length from RSA simulation with the three different parameterization scenarios. For each scenario, examples of two simulated root architectures of column-grown mature root systems are depicted, showing a genotype with a large (*Estonia3*) and small (*Latvia4*) root system, respectively. In all cases, the model-based prediction is presented as linear regression with zero intercept (see Table 7 demonstrating non-significance of the intercept) and compared with a 1:1 line. Details on the statistical accuracy of the model are given in Table 7.

**Table 7.** Statistical indicators for the goodness of fit of mature root system prediction via direct inference from seedling screens (cf. Fig. 5) and using root architecture simulation (cf. Figs 7 and 8) RMSE is root mean square error; correlation gives the Spearman rank correlation coefficient with *P*-values; slope=1:1 and intercept=0 indicate if slope and intercept parameters of a linear regression are significantly (*P*-values) different from the 1:1 line and from zero (no intercept) respectively.

Indicators	Direct comparison	Model scenario 1	Model scenario 2	Model scenario 3
**Root length**				
RMSE (cm)	565.6	591.8	839.2	614.8
Mean error (%)	99.0	19.3	29.8	23.8
Index of agreement (–)	0.41	0.94	0.51	0.95
Correlation (*R*_Spearman_; *P*-value)	0.42(*P*=0.099)	0.83 (*P*<0.001)	0.70 (*P*=0.002)	0.85 (*P*<0.001)
Slope=1:1 (*P*-value)	<0.001	0.358	0.815	0.090
Intercept=0 (*P*-value)	0.123	0.115	0.507	0.455
**First-order lateral root number**				
RMSE (*n*)	5.07	18.5	24.4	9.9
Mean error (%)	75.2	26.2	25.4	11.2
Index of agreement (–)	0.34	0.79	0.64	0.94
Correlation (*R*_Spearman_; *P*-value)	0.18 (*P*=0.497)	0.52 (*P*=0.038)	0.35 (*P*=0.178)	0.85 (*P*<0.001)
Slope=1:1 (*P*-value)	<0.001	0.002	0.028	0.555
Intercept=0 (*P*-value)	0.059	0.004	0.081	0.137
**Root depth distribution**				
RMSE (–)	–	0.18	0.18	0.10
Mean error (%)	–	71.2	73.8	38.5
Index of agreement (–)	–	0.52	0.47	0.92
Correlation (*R*_Spearman_; *P*-value)	–	0.25 (*P*=0.025)	0.20 (*P*=0.084)	0.74 (*P*<0.001)
Slope=1:1 (*P*-value)	–	<0.001	<0.001	0.001
Intercept=0 (*P*-value)	–	0.005	0.002	0.233

Scenario 1 (Fig. 7a) shows the results of mature root system prediction—using simulations with parameterization based on tap and lateral elongation functions as well as lateral root branching distance of seedlings. The slope of a linear regression (0.88; *R*^2^=0.95) is not significantly different from the 1:1 line, indicating a reliable simulation-based prediction of mature root length from seedling root parameters.

The results of Scenario 2—including measured final lengths of tap and lateral roots to parameterize an elongation function rising exponentially to these measured maxima—are shown in Fig. 7b. The slope of the linear regression (*R*^2^=0.92) is 0.83, indicating a slightly higher underestimation of the observed root length.

Figure 7c depicts the result of Scenario 3, which is equal to Scenario 1 in regard to elongation functions, but includes a scaled interbranch distance of laterals along the tap root from final measurements to test for the importance of improved branching information. The predicted absolute values are closest to the 1:1 line, with a slope of the linear regression (*R*^2^=0.95) equal to 1.05. Thus, predicting the mature root length slightly improved when using branching density information from later ontogenetic stages compared with utilizing seedling root data only (Scenario 1).

#### Simulation-based prediction of root architectural characteristics

**Fig. 8.**
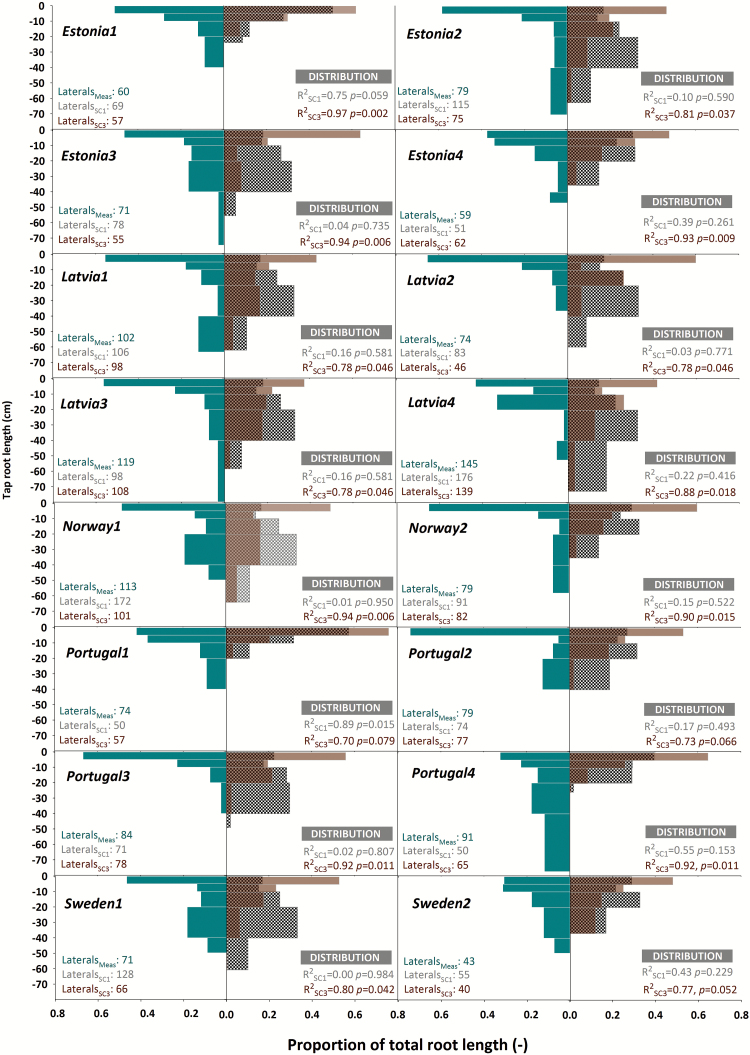
Architecture of measured and simulated pea root systems. Bars show the percentage distribution of root length along the tap root (measured, towards the left side; simulated, towards the right side with dotted bars for Scenario 1 and filled bars for Scenario 3). *R*^2^ with *P*-values for measured versus simulated distribution and number of first-order laterals is indicated for each genotype. (This figure is available in colour at *JXB* online.)

Figure 8 shows root length distribution along the tap root for Scenarios 1 (seedling root data only) and Scenario 3 (improved interbranch distance information) compared with the measured mature root systems. Scenario 2 with the same lateral branching distance as Scenario 1 is not shown graphically.

The measured root systems had an average of 51.3% of total root length in the top 5 cm, decreasing exponentially to 1.7% in the lowest 5 cm of the tap root. Genotype *Latvia2* allocates the highest proportion of root length in the top 5 cm (65.4%), while *Portugal4* has the highest allocation below 40 cm of tap root length (11.8%). The decreasing pattern is not reproduced accurately by simulation Scenarios 1 and 2 with even spacing of lateral branches along the tap root. Here the decrease follows a linear pattern from an average of 25.7% (Scenario 1) and 22.5% (Scenario 2) in the top 5 cm to 3.6% and 0.8% in the lowest 5 cm, respectively. Scenario 3, in contrast, accurately follows the observed depth distribution with an exponential trend decreasing from an average of 53.8% in the top 5 cm and 0.9% of total root length in the last 5 cm of the tap root.

The relationship between observed and simulated root length distribution along the tap root, shown in Fig. 8, is very close for Scenario 3 with an *R*^2^ between 0.97 (*Estonia1*) an 0.70 (*Portugal1*) and an average *R*^2^ of 0.85. For Scenarios 1 and 2, in contrast, the simulations do not provide a reliable prediction of root distribution: in Scenario 1, *R*^2^ ranges from the highest value of 0.89 in *Portugal1* to an *R*^2^ of <0.01 in *Sweden1* and an average of 0.25; for Scenario 2, the respective values are 0.29 for *Sweden2*, <0.01 for *Norway1*, and an average of 0.12.

Figure 8 also shows that the final tap root length is generally not reproduced accurately by the simulation Scenarios 1 and 3 (*R*^2^=0.02), in contrast to Scenario 2 (*R*^2^=0.68). However, as suggested by the measurements (see Fig. 4b), tap root length has only a minor influence on final root length. In contrast, lateral root number appears to be a key parameter for predicting both root length (see interbranch distance in Fig. 4b) and distribution. Total lateral root number (measured and simulated values are given in Fig. 8) is best predicted by Scenario 3 (*R*^2^=0.86), followed by Scenario 1 (*R*^2^=0.52), and worst by Scenario 2 (*R*^2^=0.16).

#### Statistical model evaluation

Table 7 provides statistical indicators for the prediction of mature plant root parameters (total length, lateral numbers, and length distribution) from seedling root phenotyping information, derived either by direct comparison (linear regression of seedling and mature root length; see Fig. 4) or by model-based up-scaling according to the three selected scenarios (see Figs 7, 8).

All statistical indicators confirm that model-based predictions are clearly superior to direct linear inference from seedling root data to mature root systems. Following breeders’ logic of selecting superior genotypes from a sample by allocating them into, for example, four quartiles from best to worst trait expression (highest to lowest root length), 87.5% are found in a different quartile when ranking according to seedling versus mature-stage phenotyping; in 56.2% they even shift over more than one quartile. In the case of simulation-based extrapolation, only 43.7% (Scenario 1; 56.3% for Scenario 2 and Scenario 3) change their quartile rank and only 6.3% (one of 16 genotypes) shift over two quartiles.

When using more quantitative evaluation statistics, the best prediction in both overall root system size (total length) and architectural traits (lateral numbers, depth distribution) is provided by using a linear root elongation model with phenological constraints and including scaled branching following observations from the mature ontogenetic stage (Scenario 3). This scenario results in the best prediction of genotype ranks (Spearman correlation coefficient), and also absolute values are predicted very accurately with regression slopes close to the 1:1 line and intercepts non-significantly different from zero. Although the improvement of Scenario 3 over Scenario 1 (with seedling root information only) is small in terms of total root length prediction, inference on the underlying architecture requires improved branching information. Scenario 2 with an elongation function rising exponentially to the known final root length does not provide an advantage over the linear function with phenological constraint in spite of the more accurate reproduction of the observed tap root length.

## Discussion

Phenotyping is a rapidly advancing field of plant sciences owing to technological progress in imaging capacity. This is expected to accelerate crop improvement by directly targeting traits relevant for yield potential and stress adaptation. Key requirements on phenotyping in a breeding context are (i) high throughput and (ii) reliable inference on crop performance under *in situ* conditions, namely growing in field soil over an entire vegetation cycle (Passioura, 2012; Cobb *et al.*, 2013; Walter *et al.*, 2015). These requirements are particularly critical for roots. Breeders’ ignorance of roots has been partially due to the unresolved measurement bottleneck when facing larger screening populations growing in field soil where non-invasive approaches are not feasible. Therefore, expectations are high that novel root phenotyping platforms can overcome this gap.

Most root phenotyping platforms use lab-based approaches with plants growing over short periods of time on artificial media to facilitate imaging (Clark *et al.*, 2013; Atkinson *et al.*, 2015). Shovelomics is an exception of field-based root phenotyping with comparatively high-throughput—targeting branching traits in the topsoil (Trachsel *et al.*, 2011; Colombi *et al.*, 2015). In between high-throughput platforms and traditional labour-intensive field methods (e.g. Böhm, 1979), the use of soil-filled columns or rhizoboxes allows for mature root system assessment in (semi-)natural growth media under greenhouse conditions (Vadez *et al.*, 2008; Nagel *et al.*, 2012). These systems show good agreement with field-grown plants (Kashiwagi *et al.*, 2006), but are still restricted in throughput. For example, the gel-based phenotyping system ‘GrowScreen-Agar’ used in our study allowed multiple-trait root architectural assessment of all 16 genotypes in 12 replicates within <2 weeks of experimental duration. The column system with 128 PVC tubes involved not only longer experimental duration, but substantially higher work load in set-up, handling of the system, and parameter acquisition.

Still genotype differentiation in high-throughput seedling root platforms (e.g. Ruta *et al.*, 2010; Hamada *et al.*, 2012) is challenged when pointing to the inference problem beyond the platform environment in terms of growth media and ontological stage. Hargreaves *et al.* (2009) and Wojciechowski *et al.* (2009) demonstrated a significant G×E interaction for wheat and barley root length (i.e. ranking among genotypes changed with the phenotyping set-up). Our results for pea confirmed this problem of platform-specific ranking among genotypes for all traits measured in both systems (tap and lateral root length, interbranch distance). In contrast, Watt *et al.* (2013) found a good relationship between root traits of young (two-leaf stage) wheat plants phenotyped on germination paper and field-grown plants at the same growing stage. However, in their study, the main inference problem was towards later growing stages (flowering): the seedling root observations did not show any correlation with mature root systems.

However, genetic evidence of lab-measured early root traits as relevant drivers for yield of field-grown crops (Tuberosa *et al.*, 2002) suggests that early-stage root phenotyping data should still contain breeding-relevant information for whole-plant performance. We therefore hypothesized that the frequent lack of correlation between seedling and mature root systems is only partially related to a G×PLATFORM interaction, while oversimplified empirical extrapolation methods can be a common reason for failed attempts at root phenotype prediction beyond a given experimental situation. Our data did not suggest any empirical function that reliably relates the measured root traits at different stages. The difficulty of empirical up-scaling from early to late ontogenetic stages is also underlined by the change of tap root length versus interbranch distance as drivers for lateral root length: at early stages, tap root elongation constrains lateral length, while later the distance between branching points along the tap root becomes the main constraint. We therefore suggested that extrapolation between ontological stages requires taking the biological logics of root growth and development into account.

**Fig. 9.**
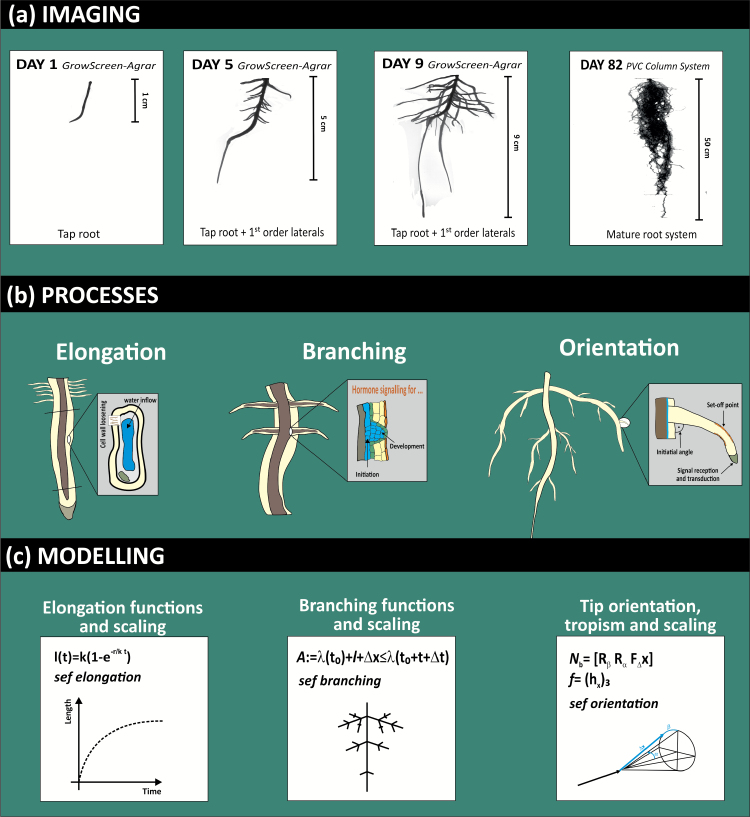
From observation to simulation. (a) Image series of a pea root system at different stages; (b) major processes involved in growth and development towards a mature root system, and (c) their tentative representation in a root architectural model. (This figure is available in colour at *JXB* online.)

Simulation models are mathematical tools to translate these biological fundaments into algorithms to capture root formation and functioning over time under different boundary conditions. Root architecture at a given time is the result of processes governing growth, branching, and orientation of root axes of different order (Fig. 9).

Root system size as expressed by the cumulative root length at a given ontogenetic stage is first of all governed by the elongation rate of single axes. Elongation involves (i) cellular processes of division, expansion, and differentiation (Croser *et al.*, 1999; Ubeda-Tomás *et al.*, 2012); (ii) phenologically changing whole-plant source–sink relations (Lemoine *et al.*, 2013); and (iii) short-term adaptive responses to environmental signals, such as via osmotic adjustment and cell wall loosening (Hsiao *et al.*, 1976; Davies and Zhang, 1991). The RootBox model used in our study sums up cellular processes into a global elongation function of single axes. Growth functions used in plant sciences most frequently involve a pre-set maximum, while differing in the shape to arrive at this end-point, such as logistic or sigmoidal growth (e.g. Morris *et al.*, 1992; Yin *et al.*, 2003). However, considering short-term elongation dynamics, root axes follow a linear pattern (Croser *et al.*, 1999). In our study, seedling plants elongated at an average rate of 1.04 cm d^–1^ (tap) and 0.71 cm d^–1^ (laterals). Azam *et al.* (2013) reported values of 2.04 cm d^–1^ with temperatures higher compared with our experiment (30 °C versus 22 °C).

Using linear elongation over longer time spans (vegetation period), it is imperative to include a reduction function that takes into account changing assimilate allocation towards the roots. As shown by Thorup-Kristensen (1998) for different pea genotypes, root growth ceases around flowering. This temporal pattern was implemented into the model using a scaling function that progressively reduces elongation with the onset of flowering. Interestingly, the resulting scaled linear elongation function performed substantially better in predicting mature root length compared with a logistic growth function with a pre-set final length from measurements. The logistic Scenario 2 was substantially less accurate in predicting the number of laterals, which is a key driver of root length (see Fig. 4b), in spite of a more accurate representation of tap root length and the same interbranch distance as in Scenario 1. This is explained by the mathematical implementation of the logistic growth function: maximum tap length results from multiplying interbranch distance by the number of laterals along the tap root and adding apical and basal lengths. Using measured interbranch distances and final tap lengths, a calculated number of laterals (*nob*) is prescribed to parameterize the growth function (see Table 6). This obviously induced a source of error in the simulation of lateral numbers; for example, in three cases, prescribed *nob* restricted lateral number formation.

The constrained linear elongation approach (Scenarios 1 and 3) thus offers three advantages: (i) better agreement between simulated and measured root length by avoiding mathematical constraints in root number simulation (*nob*); (ii) functional consideration of the biological process of changing assimilate allocation driving growth termination at a given phenological stage; and (iii) prediction of mature root system size without the necessity of a previously known maximum axes length.

Root branching is a second main process determining the overall root system size. Lateral roots originate from mature non-dividing pericycle cells of the parent root, triggered to become lateral root founder cells that undergo cell division, elongation, and differentiation (Malamy and Ryan, 2001; Aloni *et al.*, 2006). Environmental stimuli strongly influence whether a lateral root will emerge from a branching point or not (Nibau *et al.*, 2008). Facing the complex regulation of lateral branching (Atkinson *et al.*, 2014), using a fixed branching density in an RSA model is an oversimplification of the architectural diversity of root systems. Including a scaling function, however, can describe uneven lateral branching. The approach is flexible and allows scaling the branching probability following empirical data or via a submodel responding to, for example, phenology, environmental signals such as local nutrient patches (López-Bucio *et al.*, 2003), or mechanical resistance (Tsegaye and Mullins, 1994).

Spacing between lateral branches along the tap root increased from an average of 0.53 cm to 0.79 cm between seedling and mature plants. Similar values were found by other authors (e.g. Tricot *et al.*, 1997, ~0.5 cm; Rameau *et al.*, 2002, 0.52–0.81 cm). In a review focusing on Arabidopsis, Dubrovsky and Forde (2012) demonstrated that lateral root spacing within the branching zone is rather constant over plant age when considering both lateral roots and lateral root primordia. Pagès and Pellerin (1994) and Ito *et al.* (2006), in contrast, described a linear increase in branching distance of first-order laterals from the base to the tip of the tap root in mature field-grown maize, similar to our observations in mature pea roots. When excluding the lowest tap root segment (40 cm to tip) with an unknown length of the unbranched apical zone and non-emerged primordia density, the interbranch distance of genotypes linearly increased with an *R*^2^ between 0.85 and 0.99. The resulting lower number of laterals emerging from more apical parts of the tap root compared with basal segments might be one reason for the lack of influence of tap root length on total lateral length in mature genotypes (see Fig. 4b).

While mean interbranch distance from early-stage phenotyping is sufficient to predict mature root length (Scenario 1), the spatial pattern is of high importance to predict accurately the architectural shape such as depth distribution. An empirical scaling function (e.g. linear increase with tap length) could be a sufficient representation in some species such as pea in our study and maize (Pagès and Pellerin, 1994). In other species, a more constant interbranch distance has been reported (e.g. wheat; Ito *et al.*, 2006), while detailed analysis of Arabidopsis, including lateral root primordia, did not suggest any regular pattern easily represented by an empirical function, pointing to the need to model the scaled branching in response to environmental stimuli (Dubrovsky *et al.*, 2006).

When aiming to predict the spatial pattern of soil exploration in natural field conditions, further parameters would be required for architectural predictions, such as the initial branching angle of laterals from their parent root and subsequent tropic responses (Evans, 1991; Mullen and Hangarter, 2003). Although included in the RootBox model, we have not considered root orientation and tropism in this study because information on root angles was only available from the seedling platform. Phenotyping is able to capture the basic branching skeleton underlying distinct root system architectures. This can be considered as the fundamental structural predisposition of a root system for field soil exploration, where it is then further modified by responding to environmental signals. Understanding and modelling these ‘secondary’ root architectures resulting from the modified ‘primary’ root structural skeleton is a focus of intense physiological research (e.g. Palme and Teale, 2013) and studies on root–soil interactions (e.g. Dunbabin *et al.*, 2013), which, however, is beyond the scope of phenotyping.

The high prediction accuracy achieved in our study suggests an important place for RSA model application within the plant phenotyping pipeline to bridge between platforms and extrapolate beyond experimental boundaries. Elongation and branching patterns provide sufficient input data for model parameterization and allow estimation of root system formation over different ontological stages. Thereby a validated model can add high value to high-throughput phenotyping platforms by scaling up early-stage observations to breeding-relevant mature systems for subsequent *in vivo* and/or *in silico* testing of root functionality in target environments.

### Conclusions

Seedling root phenotyping allows high-throughput screening of plant material. The utility for crop improvement, however, requires reliable inference towards the mature root system. The direct relationship among traits phenotyped at different ontological stages is frequently lacking. Using a set of pea genotypes, we demonstrate that RSA models such as RootBox can overcome the apparent lack of correlation, taking into account the biological rules of root system formation within the model algorithms. Thereby RSA models provide a mechanistic extrapolation tool beyond the experimental boundaries. Our study proves that easily accessible root elongation and branching traits from an agar-based phenotyping platform are sufficient for model parameterization to predict the ranking of fully developed root systems. We demonstrate that root branching information is of particular importance to reproduce accurately distinct root architecture underlying a given root system size. We conclude that RSA models are an integral part of a phenotyping pipeline translating high-throughput early-stage traits into mature root system predictions. Although further studies with mature root observation systems are necessary for validation, there is strong evidence that root models can largely overcome the inference problem of seedling root platforms. Breeding will profit from reliable prognosis of fully developed root systems originating from seedling plants to better exploit their potential in resource acquisition and yield.

## Author contributions

BR and KN designed the study. JZ, BR, and KN performed the phenotyping experiments. AN, DL, and GB made the simulations. GB and AN evaluated the data and wrote the draft of the manuscript. BR, JZ, and KN contributed substantially to revisions.

## Supplementary Material

Supplementary Data

## Abbreviations:

DAG: day after germination
RSA: root system architecture.
