# Supplementary material for: Root architecture simulation improves the inference from seedling root phenotyping towards mature root systems
Source: J Exp Bot. 2017 Feb 7;68(5):965–82. doi: 10.1093/jxb/erw494 (PMC5441853; doi:10.1093/jxb/erw494)
Supplement: Supplementary Data [file erw494_Supplementary_Data.zip › supplementary_protocol_S1.pdf]

Root architecture simulation improves the inference from seedling root phenotyping towards mature root systems

Jiangsan Zhao, Gernot Bodner, Boris Rewald, Daniel Leitner, Kerstin A. A. Nagel, and Alireza Nakhforoosh

SUPPLEMENTARY PROTOCOL S1.

Example Matlab code for the simulation of pea genotype Estonia3

This code can be run in Matlab with the RootBox model that is freely available for download at: http://www.csc.univie.ac.at/rootbox/
For further information and in case of problems when running the model please contact: gernot.bodner@boku.ac.at

```
%========================================================================
%EXAMPLE PARAMETERIZATION FOR THE SIMULATION OF PEA GENOTYPE ESTONIA3

%Current parameters are for simulating Scenario 1;
%Parameters for Scenario 2 and Scenario 3 are given as comments to the
respective
%code line with note SC2 and SC3

%To obtain root length distribution along the tap root, the root system has
%to be streched like an antenna. This is done by setting (1) setting sigma of
tap =
%0 %(Code line 34) and sigma of lateral = 0 (code line 77), (2)theta of the
lateral = pi/2 (Code 65)
%and (3) removing the pot boundary (Code line 191).
%========================================================================

clear all;

p = cell(4,1);

%-----------------------------------------
%PARAMETERS FOR SINGLE ROOT ORDERS
%-----------------------------------------

% Tap root
%-----------------------------------------

% Parameters editable by the GUI [mean, sd]
p{1}.r = [1.25, 0]; % Initial elongation rate (cm/day) SC2: 1.10
p{1}.a = [0.07, 0]; % Root radius (cm)
p{1}.lb = [0.5, 0]; % Length of basal zone (cm)
p{1}.la = [2.5, 0]; % Length of apical zone (cm)
p{1}.ln = [0.71, 0]; % SC3: 0.13
p{1}.nob = [1000, 0]; % SC2: 105

% Additional parameters (predefined values, not altered by GUI)
p{1}.name = 'Tap root'; % Name of the root type
p{1}.color = [0.5, 0, 0]; % Color of the root (rgb)
```

```
type = 1; % Type of tropism (0 plagio-, 1 gravi-, 2 exo-, 3 chemo-
/hydrotropism)
N = 1.5; % Strength of tropism
sigma = 0.2; % Expected change of root tip heading (rad/cm) DIST: 0
p{1}.tropism = [type, N, sigma]; % Root tropism
p{1}.dx = 0.25; % Spatial resolution along root axis (cm)
p{1}.rlt = [Inf, 0]; % Maximal root life time [mean, std] (days)
p{1}.gf = 2; % Type of growth function (1: negative exponential, 2: linear)
SC2: 1

% Preset by GUI
p{1}.theta = [0, 0]; % Insertion angle(rad)
p{1}.successor = [2, 1]; % Laterals [type,probability;type,probability;...]

%Growth reduction: SC2: Comment lines 44-46
startt = 38;
ddt = 6.3;
mins = 0.0;
p{1}.sef = @(x) growthReduction(x,startt,ddt,mins); % SC2: sef = @(x) 1;

% %Branching reduction: SC3: Uncomment lines 55-58
% lb = 0.5;
% lbr = 72.1; % Tap length minus unbranched apical length set at 2.5 cm
% z_ = [0,0.03, 0.07, 0.14, 0.28, 0.55, 1]; % segments along tap root
% s_  = [1,0.64,0.24,0.08,0.05,0.01]; % percentage reduction of branching
probability
p{1}.sbpf =  @(x) 1; %SC3: sbpf = @(x) scaleBranchingFun(x,lb,lbr,z_,s_);

p{1}.saf = @(x) 1; % Scale lateral branching angle function


% First order Laterals
%-----------------------------------------

% Parameters editable by the GUI [mean, sd]
p{2}.r = [0.62, 0]; % Initial elongation rate (cm/day) SC2: 0.35
p{2}.a = [0.038, 0]; % Root radius (cm)
p{2}.theta = [0.9, 0]; % Insertion angle (rad) DIST: pi/2
p{2}.lb = [0.5, 0]; % Length of basal zone (cm)
p{2}.la = [2.5, 0]; % Length of apical zone (cm)
p{2}.ln = [0.71, 0]; % Length between laterals (cm) %
p{2}.nob = [1000, 0]; % Maximal number of laterals (1) SC2: 19

% Additional parameters (predefined values)
p{2}.name = 'First order lateral'; % Name of the root type
p{2}.color = [0, 1, 0]; % Color of the root (rgb)
type = 1; % Type of tropism (0 plagio-, 1 gravi-, 2 exo-, 3 chemo-
/hydrotropism)
N = 1; % Strength of tropism
sigma = 0.3; % Expected change of root tip heading (rad/cm) DIST: 0
p{2}.tropism = [type, N, sigma]; % Root tropism
p{2}.dx = 0.25; % Spatial resolution along root axis (cm)
p{2}.rlt = [Inf, 0]; % Maximal root life time [mean, std] (days)
p{2}.gf = 2; % Type of growth function (1: negative exponential, 2: linear)
SC2: 1
```

```
% Preset by GUI
p{2}.successor = [3, 1]; % Laterals [type,probability;type,probability;...]

%Growth reduction: SC2: Comment lines 91-93
startt = 38;
ddt = 6.3;
mins = 0.0;
p{2}.sef = @(x) growthReduction(x,startt,ddt,mins); % SC2: sef = @(x) 1;

p{2}.sbpf = @(x) 1; % Scale lateral branching probability function
p{2}.saf = @(x) 1; % Scale lateral branching angle function


% Second order laterals
% ----------------------------------------
%
% Parameters editable by the GUI [mean, sd]
p{3}.r = [0.62, 0]; % Initial elongation rate (cm/day) SC2: 0.35
p{3}.a = [0.022, 0]; % Root radius (cm)
p{3}.theta = [0.9, 0]; % Insertion angle (rad)
% p{3}.lb = [0.1, 0]; % Length of basal zone (cm)
p{3}.la = [0.5, 0]; % Length of apical zone (cm)
% p{3}.ln = [0.175, 0]; % Length between laterals (cm)
% p{3}.nob = [5, 0]; % Maximal number of laterals (1)

% Additional parameters (predefined values)
p{3}.name = 'Second order lateral'; % Name of the root type
p{3}.color = [0, 0, 1]; % Color of the root (rgb)
type = 1; % Type of tropism (0 plagio-, 1 gravi-, 2 exo-, 3 chemo-
/hydrotropism)
N = 1; % Strength of tropism
sigma = 0.3; % Expected change of root tip heading (rad/cm)
p{3}.tropism = [type, N, sigma]; % Root tropism
p{3}.dx = 0.25; % Spatial resolution along root axis (cm)
p{3}.rlt = [Inf, 0]; % Maximal root life time [mean, std] (days)
p{3}.gf = 2; % Type of growth function (1: negative exponential, 2: linear)

% Preset by GUI
p{3}.successor = [4, 1]; % Laterals [type,probability;type,probability;...]

%Growth reduction: SC2: Comment lines 127-129
startt = 38;
ddt = 6.3;
mins = 0.0;
p{3}.sef = @(x) growthReduction(x,startt,ddt,mins); % SC2: sef = @(x) 1;

p{3}.sbpf = @(x) 1; % Scale lateral branching probability function
p{3}.saf = @(x) 1; % Scale lateral branching angle function


%----------------------------------------
% Third order laterals
% NOT INCLUDED IN SIMULATION SCENARIOS
% ----------------------------------------
```

```
%-----------------------------------------
%GENERAL SETTING
%-----------------------------------------

plantingdepth = 0; % (cm)
basal_first = [Inf, 0]; % First occurence [mean, std] (days)
basal_delay = [0, 0]; % Interim time [mean, std] (days)
basal_max = 100; % Maximal number of basal roots (1)

%
%-----------------------------------------
%SIMULATION SETUP
%-----------------------------------------

% Parameters (editable by the GUI)
simtime = 82;  % simulation time (days)
dt = 1; % temporal resolution, simtime/(intermediate+1) (days)

% plant pot geometry
r1=5;
r2=5;
h=100;
pot = @(p) distPot(p,r1,r2,h);

% Initialize
p = completeParameters(p,pot); % DIST: p = completeParameters(p) %this removes
the pot boundaries
set(0,'RecursionLimit',5000); % sometimes needed
str = createDicotRS(plantingdepth,basal_first,basal_delay,basal_max); % create
initial string
N = round(simtime/dt); % number of iterations


X = [-inf, inf]; % no limits
Y = [-inf, inf]; % no limits
Z = [-inf,-40,-20,-10,-5,0]; % DIST: this evaluates the root length in the
given layers
T = [1.5, inf]; % all, e type 1

% Run simulation
for i = 1 : N

    disp(i);
    str = applyRules(str,dt); % l-system string representing the root system

    %------------------------------------------------------------
    %OUTPUT OPTIONS
    %------------------------------------------------------------


    %Output of the number of first order laterals along the tap root
        [lines,radii,colors,times,types,indS,indE] = getPolylines(str);
    o1roots(i) = sum(types==2); % order 1 roots have type==2
```

```
    %Output of DIST;
    layers(i,1:5) = squeeze(getDensity(str,X,Y,Z,'l'));

    res{i} = str; % save intermediate results


end

%-----------------------------------------
% VISUALIZATION
%-----------------------------------------

figure;
plotTubes(res{end});
% X_ = linspace(-r1,r1,100);
% Y_ = linspace(-r1,r1,100);
% Z_ = linspace(-h,0,40);
% plotDistFunc(pot,X_,Y_,Z_,[0 0 1]); % approximate the pot boundaries
% view(3);



%-----------------------------------------
% EVALUATIONS
%-----------------------------------------

v = zeros();
for i = 1 : N
    v(i) = getTotal(res{i},'l'); % or 's' for surface, see getTotal
end
figure;
plot((1:N).*dt,v);
xlabel('Time (days)');
ylabel('Root system length (cm)');


hold on;
plot((1:N).*dt,layers);

legend('total lenght','length in [-40..-inf]','length in [-10..-20]','length
in [-10..-20]','length in [-10..-5]','length in [-5..0]')


% Analysis of lateral z-positions

%Define depth for evaluation
lb =  p{1}.lb(1);
lbr = (o1roots(82)-1)*p{1}.ln(1);
z_ = [0,0.20,0.40,1]; % relative depth

[lines,radii,colors,times,types] = getPolylines(res{end});
c=1;
for i = 1 : length(lines)
    if types(i)==2 % laterals
        x = lines{i};
```

```
        zl(c) = x(1,3); % 1 start node, 3 z..coord.
        c=c+1;
    end
end

lnZ = mean(abs(diff(zl))); % mean z inter-lateral distance

zs = max(min((abs(zl)-lb)/lbr,1),0); % scaled from 0 - 1

for i =  2 : length(z_) % mean z inter-lateral distances per zone z_
    I = zs>=z_(i-1) & zs<z_(i);
    lnZ_(i) = mean(abs(diff(zl(I))));
end



bnd = getBounds([],lines);
% root length fraction / depth
X = [-inf inf]; Y = [-inf inf];
zz = -z_*lbr-lb;
Z = [bnd(5),zz(end:-1:1),0];
[map,l] = getDensity(str,X,Y,Z,'l'); % l for lenght, s for surface
d = squeeze(map);
figure; hold on;
for i = 1 : length(d)
    plot([d(i) d(i)],[Z(i+1),Z(i)],'b');
end
ylabel('depth (cm)');
xlabel('root length (cm)');
```
