# Supplementary material for: Root architecture simulation improves the inference from seedling root phenotyping towards mature root systems
Source: J Exp Bot. 2017 Feb 7;68(5):965–82. doi: 10.1093/jxb/erw494 (PMC5441853; doi:10.1093/jxb/erw494)
Supplement: Supplementary Data [file erw494_Supplementary_Data.zip › supplementary_table_S1.pdf]

Root architecture simulation improves the inference from seedling root phenotyping towards mature root systems

Jiangsan Zhao, Gernot Bodner, Boris Rewald, Daniel Leitner, Kerstin A. A. Nagel, and Alireza Nakhforoosh

Supplementary Table S1.

First order lateral root branching information for interbranch distance scaling

Table S1 gives the detailed interbranch distance data of first order laterals along the tap root measured in the mature phenotyping system. These data are used to parameterize a scaling function (sbf) in the root architecture model RootBox.

For this purpose the branching zone l_n of the tap root (total tap length minus the unbranched zones at the base l_b and the apex l_a) is subdivided into different segments (in % of the branching zone length). The interbranch distance for each segment $l_{ns,i}$ is obtained via a branching probability calculated by dividing the interbranch distance of the first segment (0-2.5 cm) by the interbranch distance in the respective segment (i.e. $l_{n2.5}/l_{ns,i}$).

Table S1. Interbanch distances in different segments along the tap root.

**Genotype**	***0-2.5cm***	***2.5-5 cm***	***5-10 cm***	***10-20 cm***	***20-40 cm***	***40-tap end****
Estonia1	0.16	0.21	0.35	0.76	3.87	28.25
Estonia2	0.13	0.18	0.42	0.65	1.63	5.53
Estonia3	0.12	0.18	0.48	1.54	2.14	8.67
Estonia4	0.24	0.25	0.35	0.89	2.46	-
Latvia1	0.17	0.15	0.25	0.71	1.36	1.05
Latvia2	0.13	0.16	0.43	0.82	0.93	-
Latvia3	0.17	0.18	0.28	0.54	0.81	1.40
Latvia4	0.10	0.12	0.21	0.38	0.91	0.45
Norway1	0.11	0.16	0.45	0.49	0.82	0.53
Norway2	0.12	0.18	0.34	0.62	1.10	4.65
Portugal1	0.12	0.19	0.42	0.73	2.03	1.95
Portugal2	0.13	0.19	0.27	0.71	1.53	-
Portugal3	0.10	0.16	0.31	0.48	3.33	2.31
Portugal4	0.14	0.20	0.45	0.76	1.54	1.61
Sweden1	0.16	0.16	0.30	0.56	2.38	-
Sweden2	0.39	0.36	0.65	1.22	2.40	4.14

* For the length of the last segment *cf.* Tap root length in Table 5. No entry in this last segment means that no laterals emerge below 40 cm or that the tap root is shorter than 40 cm.
